# High-order scheme for the source-sink term in a one-dimensional water temperature model

**DOI:** 10.1371/journal.pone.0173236

**Published:** 2017-03-06

**Authors:** Zheng Jing, Ling Kang

**Affiliations:** School of Hydropower and Information Engineering, Huazhong University of Science and Technology, Wuhan, China; COMSATS Institute of Information Technology, PAKISTAN

## Abstract

The source-sink term in water temperature models represents the net heat absorbed or released by a water system. This term is very important because it accounts for solar radiation that can significantly affect water temperature, especially in lakes. However, existing numerical methods for discretizing the source-sink term are very simplistic, causing significant deviations between simulation results and measured data. To address this problem, we present a numerical method specific to the source-sink term. A vertical one-dimensional heat conduction equation was chosen to describe water temperature changes. A two-step operator-splitting method was adopted as the numerical solution. In the first step, using the undetermined coefficient method, a high-order scheme was adopted for discretizing the source-sink term. In the second step, the diffusion term was discretized using the Crank-Nicolson scheme. The effectiveness and capability of the numerical method was assessed by performing numerical tests. Then, the proposed numerical method was applied to a simulation of Guozheng Lake (located in central China). The modeling results were in an excellent agreement with measured data.

## Introduction

Water temperature significantly affects aquatic ecosystems. A precise forecast of the temporal and spatial variation of water temperature can help to understand the physical, chemical, and biological processes occurring in aquatic systems, and to determine suitable economic and scientific conservation strategies.

Studying the processes that affect the water temperature and thermal stratification in lakes is of especial significance. Research indicates that shallow lakes can exhibit thermal stratification lasting for several days and possibly longer [1]. Thermal stratification can lead to a series of ecological responses, such as stratified flows, differences in water density between the surface and bottom layers [2], and changes in the aquatic population structure and eutrophication processes [3]. Therefore, uncovering the processes that underlie temperature change and thermal stratification in shallow lakes can help to better understand the associated physical, chemical, and biological processes, and to develop suitable economic and scientific conservation strategies, thereby providing data and technological support for lake environmental management.

Assuming lateral uniformity of temperature in shallow lakes, the process of temperature change in lakes is usually described as a vertical one-dimensional (1D) heat conduction model. Dake and Harleman [4] developed the first 1D model to predict vertical temperature profiles in lakes or reservoirs. Later models accounted for the heat budget by computing the heat fluxes from solar radiation, convection, and evaporation at the water-air interface [5, 6]. Kim and Chapra [7] used a vertical 1D well-mixed model to predict the temperature in a shallow stream.

Development of realistic temperature models requires having suitable numerical methods for simulations of water temperature. An operator-splitting method, documented by Strang [8], uses dedicated numerical solvers for each physical phenomenon accounted by a specific model (e.g., advection, diffusion). The method has become very popular for solving many hyperbolic and parabolic equations. Valocchi and Malmstead used the operator-splitting method for discretizing the advection-dispersion-reaction equation [9]. Blom and Verwer compared four splitting methods for solving the advection-dispersion-reaction equation [10]. To address some practical needs associated with simulation approaches, many high-order schemes were developed for solving computational fluid dynamics (CFD) equations, such as the Holly–Preissmann scheme [11], the six-point scheme [12], and the WAF method [13]. Most of these methods focus on the convection and diffusion terms rather than the source-sink term. However, scholars gradually realized that an accurate discretization of the source-sink term is equally important as those of the convection and diffusion terms [14]. Siviglia and Toro noted that for inappropriately discretized source-sink terms, the overall results may be almost the same as those obtained using lower-order schemes [13]. The source-sink term in a water temperature model is very important because it accounts for the heat budget process such as solar radiation, which significantly affects the distribution of water temperature. In addition, in some temperature models, the source-sink term is more complicated, depending not only on time and space, but also on temperature. Source-sink terms of this variety may be more problematic (e.g., as in the 1D stream temperature model by Siviglia and Toro [13], in which the source-sink term depended on the stream temperature). Unfortunately, numerical methods for the source-sink term have received little attention. Existing numerical discretization methods are quite simplistic, such as the pointwise method (a simple evaluation of the source term functions at the grid point) [15]. This simplicity can lead to large deviations between simulation results and measured data. Thus, more efficient numerical methods for discretizing the source-sink term are strongly needed.

In this paper, we apply a high-order scheme for the source-sink term in a 1D vertical water temperature model to precisely predict the water temperature in shallow lakes. Using the operator-splitting method, the vertical 1D water temperature model was solved in two steps: (1) the source-sink term was discretized in the proposed high-order scheme, using an undetermined coefficient method; (2) the diffusion term was discretized using the Crank–Nicolson scheme. The proposed method was used in numerical testing, and the significance of the proper treatment of the source-sink term was assessed. Finally, the proposed method was applied to the Guozheng Lake data.

## Mathematical model

### Water temperature model

Water temperature in a shallow lake depends on the heat exchange with the atmosphere and with the bottom sediment. As a rule, small and stratified lakes exhibit weak horizontal temperature gradients, implying lateral uniformity of temperature. Thus, the process of temperature change can be described by a vertical 1D heat conduction model [16]

$$\frac{\partial T}{\partial t}=\frac{\partial }{\partial z}(K_{z}\frac{\partial T}{\partial z})+\frac{1}{\rho c_{p}}\frac{\partial I}{\partial z}$$ (1)

where *z* denotes the vertical coordinate and *z* = 0 is the surface level; *t* is time; *T* is the water temperature (°C); *K*_*z*_ is the vertical diffusion coefficient (m^2^/s); *I* is the solar radiation (W/m^2^); *ρ* is the water density (1.0 × 10^3^ kg/m^3^); *c*_*p*_ is the water specific heat capacity [4.2 × 10^3^ J/(kg°C)].

The second term on the right hand side of Eq (1), related to solar radiation, can be regarded as the source-sink term. The Beer–Lambert–Bouguer radiation model (Beer’s law) is often adopted for calculating solar radiation. The radiation is described by an exponential attenuation function. Although this approach is very popular, it requires having a significant amount of observed data. However, solar radiation data suitable for simulations are quite scarce, owing to the high associated cost of monitoring. In addition, data insufficiency requires using interpolations and averaging. While distinct from other meteorological indicators, solar radiation exhibits an obvious diurnal variation. Given the data scarcity, the obvious differences in daily and nocturnal solar radiation patterns may be eliminated after performing a simple interpolation or averaging; in either case, the interpolated (or averaged) solar radiation will be very different from the actual one, potentially yielding significant deviations of simulation results from real data. Therefore, to quantify the influence of solar radiation on water temperature in a more feasible manner, a practical method sufficiently grounded in physics is required. It is well known that a close relationship exists between solar radiation and water temperature. This relationship can be quantified by introducing a variable α that represents the ratio of water temperature to solar radiation, namely α = *T*/*I*, where *T* is the water temperature and *I* is the solar radiation. Solar radiation and water temperature are time-dependent implying that α is time-dependent as well. The time dependence of α is believed to be subject to physical principles. The relationship between solar radiation and water temperature should obey certain principles for a specified body of water (e.g., a lake) over a designated time frame (i.e., seasonal patterns). Thus, α(t) can be described using a mathematical function (e.g., a trigonometric function, a logarithmic function, or even a complicated piecewise function). Of note is that α(t) can have a more complicated form (e.g., α(t) can account for the effects of solar radiation, air temperature, and wind force). Yet this is not necessary because the effects of air temperature and wind force on water temperature have been considered in the water temperature model (as is described below when discussing the problem’s boundary conditions). The main role of α(t) is to relate water temperature to solar radiation to enable replacing the spatial gradient of solar radiation with the gradient of the water temperature. This link would allow to solve the water temperature model [Eq (1)] even in the absence of solar radiation data. Substituting α(t) into Eq (1) yields Eq (2):

$$\frac{\partial T}{\partial t}=\frac{\partial }{\partial z}(K_{z}\frac{\partial T}{\partial z})+\frac{\alpha (t)}{\rho c_{p}}\frac{\partial T}{\partial z}$$ (2)

After the substitution, the source-sink term in Eq (2) (the second term on the right hand side) can be understood as capturing the effects of solar radiation (external heating) on the temperature of water in a natural lake [13]. Although α(t) is time-dependent, the time dependence does not impact the final mathematical expression of the finite difference equation [Eq (16)]. At any time, the exact value of α can be obtained from an empirical function or a PC program. Eqs (1) and (2) appear to be similar, but are actually quite different. Eq (1) features a source term of the form *R*(*z*) (where *R* denotes the source term operator), while Eq (2) features a source term of the form *R*(*T*). In principle, *R*(*T*) might be more problematic that *R*(*z*) because it depends on the state variable (temperature). Therefore, it is not clear whether conventional numerical methods are still applicable to the system described by Eq (2). If this is not the case, then numerical methods will have to be developed for properly treating the source term in Eq (2).

### Boundary conditions

Atmospheric heat fluxes were computed from a balance between solar radiation (W/m^2^), long-wave radiation *H*_*L*_ (W/m^2^), evaporation heat flux *H*_*E*_ (W/m^2^), and convective heat transfer *H*_*C*_ (W/m^2^). Solar radiation was included in the source term while the other heat fluxes were considered at the free surface boundary condition. The heat flux at the water-sediment interface *H*_*B*_ (W/m^2^) was considered in the bottom boundary condition.

#### Boundary conditions [17]

**Water surface:**
  $$\begin{array}{cc} -\rho c_{p}K_{z}\frac{\partial T}{\partial z}=H_{L}+H_{E}+H_{C} & \left(z=0\right) \end{array}$$ (3)
**Water bottom:**
  $$\begin{array}{cc} -\rho c_{p}K_{z}\frac{\partial T}{\partial z}=H_{B} & \text{(}z=-\text{H)} \end{array}$$ (4)

The plus signs before *H*_*L*_, *H*_*E*_, *H*_*C*_ capture the fact that the heat transfers from water to air. The plus sign before *H*_*B*_ captures the fact that the heat transfers from sediment to water.

#### (a) Long-wave radiation

*H*_*L*_ was computed from Eq (5) [7]:

$$H_{L}=\varepsilon \sigma {\left(T_{s}+273.15\right)}^{4}-\varepsilon \sigma {\left(T_{a}+273.15\right)}^{4}(C_{a}+0.031\sqrt{e_{a}})$$ (5)

In Eq (5), *σ*_*w*_ is the Stefan–Boltzmann constant [W/(m^2^K^4^)], and its value is *σ*_*w*_ = 5.67 × 10^−8^; *ε* is the emissivity of the water surface, set to *ε* = 0.97; *T*_*a*_ is the air temperature (°C); and *C*_*a*_ is the coefficient related to the air temperature, set to *C*_*a*_ = 0.6 in this paper.

#### (b) Evaporation heat flux

Water evaporation is the major route of heat loss for a lake. The evaporation-related heat flux *H*_*E*_ was computed from Eqs (6–8) [18, 19]:

$$H_{E}=f(w)(e_{s}-e_{a})$$ (6)

$$f(w)=(a_{0}+a_{1}w+a_{2}w^{2})$$ (7)

$$e_{s}=6.112\text{exp}(\frac{17.67T_{a}}{T_{a}+243.5})$$ (8)

In Eqs (6–8), *e*_*s*_ is the saturated vapor pressure (mb); *e*_*a*_ is the vapor pressure (mb), *e*_*a*_ = hum*e_*s*_, *hum* is the air humidity (%); *w* is the wind speed (m/s). Some expressions are found in the literature for the wind speed function *f*(*w*) [7, 20]. Ahsan and Blumberg discussed the form of *f*(*w*) [21]. The correlation used by Ahsan and Blumberg [21] was adopted in this paper (*a*_*0*_ = 6.9, *a*_*1*_ = 0 and *a*_*2*_ = 0.345).

#### (c) Convective heat transfer

Sensible heat conduction corresponds to the heat exchange between water and the atmosphere, driven by a temperature gradient. Sensible heat conduction is mainly caused by conduction and convection. In the present work, *H*_*C*_ was computed from Eq (9) [22, 23]:

$$H_{c}=c_{h}\rho_{a}c_{pa}w(T_{s}-T_{a})$$ (9)

In Eq (9), *C*_*h*_ is the turbulence exchange coefficient, set to *C*_*h*_ = 1.1 × 10^−3^; *ρ*_*a*_ is the atmosphere density, *ρ*_*a*_ = 1.2 (kg/m^3^); *c*_*pa*_ is the specific heat capacity of water, *c*_*pa*_ = 1.005 × 10^3^ [J/(kg°C)]; *T*_*w*_ is the surface water temperature (°C).

#### (d) Heat flux at the water-sediment interface

The heat flux at the water-sediment interface is much weaker than that at the water-air interface; consequently, the former was often neglected in previous studies. However, evidence suggests that the heat flux at the water-sediment interface is significant when shallow lake stratifies [24, 25]. Various methods have been used for calculating the heat flux at the water-sediment interface, such as “the gradient method” [26], and “the integration of the lake sediment temperature profile” [27]. However, all of these methods still require solving complicated partial differential equations (PDEs). In this study, an equation from the CE-QUAL-W2 software was adopted for calculating *H*_*B*_ [Eq (10)]. This equation assumes that *H*_*B*_ is directly proportional to the temperature difference between the water and sediment [28].

$$H_{B}=-K_{B}(T_{b}-T_{B})$$ (10)

where *K*_*B*_ is the sediment’s heat transfer coefficient, set to *K*_*B*_ = 0.01 in this study; *T*_*B*_ is the sediment temperature (°C); and *T*_*b*_ is the water temperature at the water-sediment interface (°C).

## Numerical solution

Choosing a suitable discretization scheme is critical for obtaining a good solution. The finite difference method (FDM) was adopted for spatial discretization in this study. Since each heat flux procedure in lake temperature systems possesses distinct physical features, the most suitable discretization scheme for each operator differs from all other operators. Thus, it is difficult to select an optimal discretization scheme that would satisfy the requirements imposed by these different possesses. To overcome this problem, we used the operator-splitting method.

### Operator-splitting method

The operator-splitting method [8] allows to solve the problem imposed by the difficulty of selecting a unified discretization scheme for discretizing all operators. Although arbitrarily replacing one scheme with another (especially replacing an implicit scheme with an explicit scheme) will create some inconsistencies, research has shown that using a proper time step can help to avoid inconsistencies. Using the operator-splitting method, Eq (2) can also be formulated in the following form:

C(T)=D(T)+R(T) (11)

Where *C* is the overall (effective) operator, *D* is the diffusion operator and *R* is the source-sink operator. Therefore, the full initial value problem (IVP) in Eq (2) can be written as a system of PDEs and initial conditions (ICs):

$$\text{IVP}:\left\{\begin{array}{l} \text{PDEs}:\partial_{t}T=D(T)+R(T)\\ \text{ICs}:T(z,0)=T^{n} \end{array}\right\}\overset{\Delta t}{\Rightarrow }T^{n+1}$$ (12)

Then, Eq (12) can be divided into two parts:

$$\text{IVP}1:\left\{\begin{array}{l} \text{PDEs}:\partial_{t}T=R(T)\\ \text{ICs}:T(z,0)=T^{n} \end{array}\right\}\overset{\Delta t}{\Rightarrow }T1$$ (13)

$$\text{IVP}2:\left\{\begin{array}{l} \text{PDEs}:\partial_{t}T=D(T)\\ \text{ICs}:T(z,0)=T1 \end{array}\right\}\overset{\Delta t}{\Rightarrow }T^{n+1}$$ (14)

First, IVP1 [Eq (13)] is solved in one time step Δ*t*. It is worth mentioning that the initial condition *T*^*n*^ in IVP1 is the initial condition of the full IVP. Next, the intermediate solution *T1* is defined as the solution of IVP1, and *T1* is defined as the initial condition of IVP2. Then, IVP2 [Eq (14)] is solved, and *T*^*n+1*^ from IVP2 is output as the solution of the full IVP.

### Layout of variables

**Fig 1:**
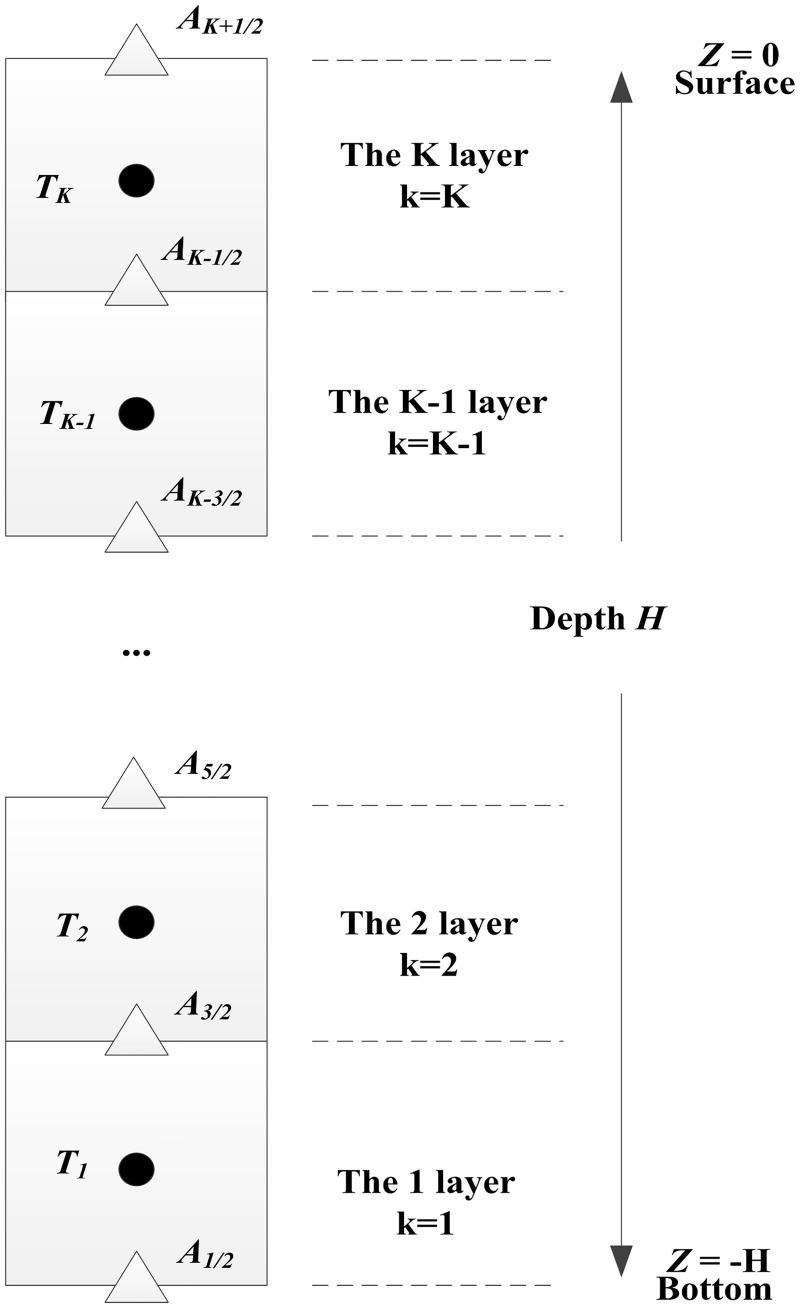
Layout of variables.

Fig 1 shows the layout of the model variables. In this figure, for better presentation, mesh cells represent water cells. A mesh cell in Fig 1 should be interpreted as a segment in the vertical direction, without a horizontal space step. Suppose water body is divided into K cells in the vertical direction. *k* denotes the mesh center in the *z* direction, with the space step of Δ*z*. The index *k* = 1 corresponds to the bottom cell, and the index *k* = K corresponds to the surface cell. Water temperature *T* locates in the middle of a cell, and is indexed as *T*_*k*_; *A* represents the upper or lower surface of a cell, and is indexed as *A*_*k*±1/2_; *H* is the overall depth of water; *z*_*k*_ is the displacement from the water surface to the center of cell *k*. The system is solved by advancing from the current time state, n, to the next time state, n+1, with the time step of Δ*t*.

### Solution of IVP1: A high-order scheme for the source-sink term

In the operator-splitting approach, the right hand side of Eq (2) is divided into two parts, namely the source-sink term operator and the diffusion operator. Considering only the source-sink term operator is equivalent to solving the following equation:

$$\frac{\partial T}{\partial t}-\frac{\alpha (t)}{\rho c_{p}}\frac{\partial T}{\partial z}=0$$ (15)

The pointwise method is often used for the temperature-dependent source-sink term [*R*(*T*)]. Yet it was claimed in some studies that this method may not work in complicated cases. This paper adopts an improved method to discretize the source-sink term. This method is based on selecting an appropriate scheme for Eq (15), e.g., a six-point scheme. To obtain a more accurate scheme, the following strategy is used: every node is given a certain weight coefficient, following which the appropriate values of these weight coefficients are determined for minimizing numerical fluctuations and diffusion. According to the undetermined coefficient method [29], the final form the finite difference equation can be written as

$$a_{1}T1_{k-1}^{n+1}+a_{2}T1_{k}^{n+1}+a_{3}T1_{k+1}^{n+1}=a_{4}T1_{k-1}^{n}+a_{5}T1_{k}^{n}+a_{6}T1_{k+1}^{n}$$ (16)

Where *a*_*i*_ (*i* = 1,2,…,6) are the undetermined coefficients. By using appropriate values of *a*_*i*_, a higher-order scheme can be obtained. Using the Taylor expansion on the grid *T*(*i*, *k*), we set $B=-\frac{\alpha \left(t=t^{n}\right)}{\rho c_{p}}$, where *t*^*n*^ represents the current time, and Eq (16) becomes:

$$\begin{array}{l} (a_{1}+a_{2}+a_{3})\Delta t\frac{\partial T}{\partial t}+(-a_{1}+a_{3}+a_{4}-a_{6})\Delta z\frac{\partial T}{\partial z}=-a_{1}-a_{2}-a_{3}+a_{4}+a_{5}+a_{6}\\ -[\left(a_{1}+a_{2}+a_{3}\right)\frac{B^{2}{\left(\Delta t\right)}^{2}}{2}+(a_{1}-a_{3})B\Delta t\Delta z+(a_{1}+a_{3}-a_{4}-a_{6})\frac{{\left(\Delta z\right)}^{2}}{2}]\frac{\partial^{2}T}{\partial z^{2}}\\ +[(a_{1}+a_{2}+a_{3})\frac{B^{3}{\left(\Delta t\right)}^{3}}{6}+(a_{1}+a_{3})\frac{B\Delta t{\left(\Delta z\right)}^{2}}{2}\text{+}(a_{1}-a_{3})\frac{B^{2}{\left(\Delta t\right)}^{2}\Delta z}{2}+(a_{1}-a_{3}-a_{4}+a_{6})\frac{{\left(\Delta z\right)}^{3}}{6}]\frac{\partial^{3}T}{\partial z^{3}}\\ -[(a_{1}+a_{2}+a_{3})\frac{B^{4}{\left(\Delta t\right)}^{4}}{24}+(a_{1}-a_{3})\frac{B\Delta t{\left(\Delta z\right)}^{3}}{6}+(a_{1}+a_{3})\frac{B^{2}{\left(\Delta t\right)}^{2}{\left(\Delta z\right)}^{2}}{2}\\ +(a_{1}-a_{3})\frac{B^{3}{\left(\Delta t\right)}^{3}\Delta z}{6}+(a_{1}+a_{3}-a_{4}-a_{6})\frac{{\left(\Delta z\right)}^{4}}{24}]\\ +\ldots \end{array}$$ (17)

Thus, Eq (17), which is derived from Eq (16), is the equivalent finite difference equation of Eq (15). By comparing Eqs (17) and (15), to minimize numerical spurious oscillations, the following algebraic equations can be obtained:

$$\begin{array}{l} a_{1}+a_{2}+a_{3}=1;\\ -a_{1}+a_{3}+a_{4}-a_{6}=\frac{B\Delta t}{\Delta z};\\ -a_{1}-a_{2}-a_{3}+a_{4}+a_{5}+a_{6}=0;\\ (a_{1}+a_{2}+a_{3}){\left(\frac{B\Delta t}{\Delta z}\right)}^{2}+2(a_{1}-a_{3})(\frac{B\Delta t}{\Delta z})+a_{1}+a_{3}-a_{4}-a_{6}=0;\\ (a_{1}+a_{2}+a_{3}){\left(\frac{B\Delta t}{\Delta z}\right)}^{3}+3(a_{1}-a_{3}){\left(\frac{B\Delta t}{\Delta z}\right)}^{2}+3(a_{1}+a_{3})\frac{B\Delta t}{\Delta z}+a_{1}-a_{3}-a_{4}+a_{6};\\ (a_{1}+a_{2}+a_{3}){\left(\frac{B\Delta t}{\Delta z}\right)}^{4}+4(a_{1}-a_{3}){\left(\frac{B\Delta t}{\Delta z}\right)}^{3}+6(a_{1}+a_{3}){\left(\frac{B\Delta t}{\Delta z}\right)}^{2}+4(a_{1}-a_{3})\frac{B\Delta t}{\Delta z}+a_{1}+a_{3}-a_{4}-a_{6}=0 \end{array}$$ (18)

Solving Eq (18) gives:

$$\begin{array}{ll} a_{1}=\frac{1}{12}(\frac{B\Delta t}{\Delta z}-1)(\frac{B\Delta t}{\Delta z}-2)\;; & a_{2}=1-\frac{1}{6}[{\left(\frac{B\Delta t}{\Delta z}\right)}^{2}+2]\\ a_{3}=\frac{1}{12}(\frac{B\Delta t}{\Delta z}+1)(\frac{B\Delta t}{\Delta z}+2)\;; & a_{4}=\frac{1}{12}(\frac{B\Delta t}{\Delta z}+1)(\frac{B\Delta t}{\Delta z}+2)\\ a_{5}=1-\frac{1}{6}[{\left(\frac{B\Delta t}{\Delta z}\right)}^{2}+2]\;; & a_{6}=\frac{1}{12}(\frac{B\Delta t}{\Delta z}-1)(\frac{B\Delta t}{\Delta z}-2) \end{array}$$ (19)

Using Eq (19), the finite difference equation [Eq (16)] for all grid points *T*(*i*, *k*) can be obtained. These equations form a system of a linear tri-diagonal matrix equations. This system can be solved using the Thomas method [30]. Finally, the intermediate temperature *T1* can be calculated. The empirical function α(*t*) can be obtained by fitting historic water temperature and solar radiation data to the model. The values of α in each time step can be calculated from the empirical function, so that the finite difference equation [Eq (16)] at that corresponding time can be obtained.

### Solution of IVP2: Crank-Nicolson scheme for the diffusion term

Using the calculated intermediate temperature *T1* from the first step, only considering the diffusion operator in Eq (2) yields the following equation:

$$\frac{\partial T}{\partial t}=\frac{\partial }{\partial z}(K_{z}\frac{\partial T}{\partial z})$$ (20)

The Crank-Nicolson scheme (which is second-order accurate in space and time) was used for treating the diffusion operator. In its discretized form, the Crank-Nicolson scheme can be expressed as follows:

$$\frac{\partial f}{\partial t}=\frac{f_{i}^{n+1}-f_{i}^{n}}{\Delta t}$$ (21)

$$\frac{\partial f}{\partial z}=\frac{1}{2}(\frac{f_{i+1}^{n+1}-f_{i-1}^{n+1}}{2\Delta z}+\frac{f_{i+1}^{n}-f_{i-1}^{n}}{2\Delta z})$$ (22)

$$\frac{\partial^{2}f}{\partial z^{2}}=\frac{1}{2}(\frac{f_{i+1}^{n+1}-2f_{i}^{n+1}+f_{i-1}^{n+1}}{{\left(\Delta z\right)}^{2}}+\frac{f_{i+1}^{n}-2f_{i}^{n}+f_{i-1}^{n}}{{\left(\Delta z\right)}^{2}})$$ (23)

The finite difference form of Eq (20) can be found in literature [13]. Using these equations, the final *T*^*n+1*^ of the whole cell can be calculated.

## Numerical tests

The proposed numerical method was applied to the numerical model, and the numerical results were compared with exact solutions for validating the method. Eq (24) (published in [13]) was selected as the test equation. This equation was selected because the source term in Eq (24) follows the source term in Eq (2), which is temperature-dependent (namely, *R = R*(*T*)). Hence, the test results have great reference value.

$$\frac{\partial T}{\partial t}=D\frac{\partial^{2}T}{\partial z^{2}}+a_{0}\text{cos}(\omega_{p}t)T$$ (24)

In the above equation, *T* is the water temperature; *D* is the diffusion coefficient; *a*_*0*_ is the amplitude; *ω*_*p*_ *= 2π/T*_*p*_; *T*_*p*_ is the period of oscillations. All of the variables are non-dimensional. The water depth was set to *H* = 4000. The boundary conditions at both interfaces were zero gradient (ə*T*/ə*z* = 0). The initial condition was

$$T(z,0)=\{\begin{array}{ll} T_{0}=10 & z_{L}<z<z_{R}\\ T_{0}=0 & z>z_{R}\text{ or }z<z_{L} \end{array}$$ (25)

With *z*_*L*_ and *z*_*R*_ set to 1500 and 1800, respectively. The initial condition prescribed a square wave between *z*_*L*_ and *z*_*R*_. Therefore, temperature was discontinuously distributed in the computational domain. Besides, the source-sink term was cosine-like. Thus, the proposed numerical experiment allowed to test whether the proposed numerical method could be used to exactly predict the thermal wave propagation in the presence of such oscillations. The numerical results were compared to the exact solution of this system, which is:

$$T(z,t)=\frac{T_{0}}{2}\left[\text{erf(}\frac{z-z_{L}}{2\sqrt{Dt}}\text{)}-\text{erf(}\frac{z-z_{R}}{2\sqrt{Dt}}\text{)}\right]\text{exp}\left[\frac{a_{0}}{\omega_{p}}\text{sin}(\omega_{p}\text{t})\right]$$ (26)

### Experiment 1: Pure diffusion

**Fig 2:**
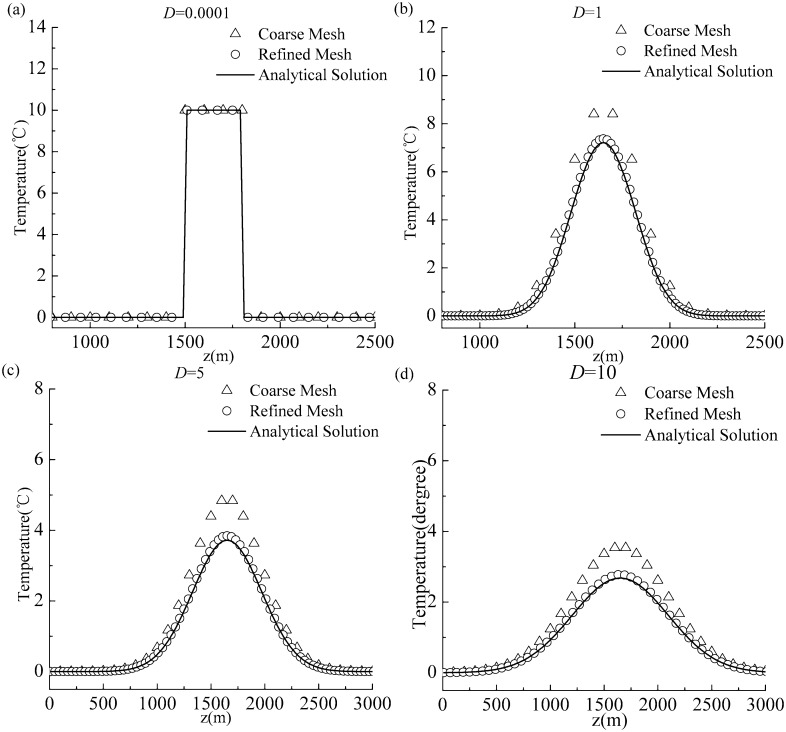
Comparison of simulation outcomes and analytical solutions for Experiment 1: (a) *D* = 0.0001; (b) *D* = 1; (c) *D* = 5; (d) *D* = 10.

Let *a*_*0*_ = 0; then, Eq (24) only contains the diffusion term. The main purpose of this experiment was to check whether the proposed numerical technique possessed the capability to exactly predict thermal wave propagation in the condition of pure diffusion. The difference scheme for the diffusion term was the Crank–Nicolson scheme. We considered different diffusion coefficients (*D* = 0.0001, *D* = 1, *D* = 5, and *D* = 10) and conducted four experiments. For each experiment, two cases were considered: (1) a coarse mesh (Δ*z* = 100) and (2) a refined mesh (Δ*z* = 10). The time step was Δ*t* = 10. The outcome at the time *t* = 9600 was chosen. The simulation results were compared to the analytical solutions and the results of this comparison are shown in Fig 2. In Fig 2(a)–2(d), circles correspond to the case of the refined mesh, and the results are presented for different diffusion scenarios; this illustrates that the solution obtained using the Crank–Nicolson scheme can satisfactorily reproduce the square wave’s location and temperature peak, with no spurious oscillations induced by sharp temperature transitions. In the case of the coarse mesh, for weak diffusion (*D* = 0.0001) [triangles in Fig 2(a)], the scheme can capture temperature discontinuities. However, for larger *D*, the numerical solutions become inconsistent with the analytical ones (especially regarding the temperature peak prediction), as is captured by triangles in Fig 2(b)–2(d). In conclusion, the results of Experiment 1 demonstrate that the Crank–Nicolson scheme is sufficiently stable and accurate for solving the pure diffusion problem. The accuracy is also closely related to the spatial step Δ*z*. Coarse spatial steps yield large truncation errors that distort the solution.

### Experiment 2: Diffusion term and source-sink term

In this experiment, we set *a*_*0*_ = 0.005 and *T*_*p*_ = 1500. The purpose of this experiment was to check whether the proposed method has the capability to predict the thermal wave propagation process in the presence of a long-lasting disturbance. Similar to Experiment 1, using the refined mesh (Δ*z* = 10), we considered different diffusion coefficients (*D* = 0.0001, *D* = 1, *D* = 5, and *D* = 10). We considered two methods for numerical simulations. The first method was the method proposed in this paper (referred to as RESOURCE1); in this method, the source-sink term was discretized using the proposed high-order scheme based on the undetermined coefficient method, and the diffusion term was discretized using the Crank–Nicolson scheme. The second method was referred to as RESOURCE2; in this method, the source-sink term was discretized using the conventional pointwise method [*R* = *R* (*T*_*k*_^*n*^)], and the diffusion term was discretized using the Crank–Nicolson scheme. The outcome at the time *t* = 9600 was analyzed. The time step was selected to maintain consistency with Experiment 1 and satisfy the Courant–Friedrichs–Lewy (CFL) condition to ensure stability.

**Fig 3:**
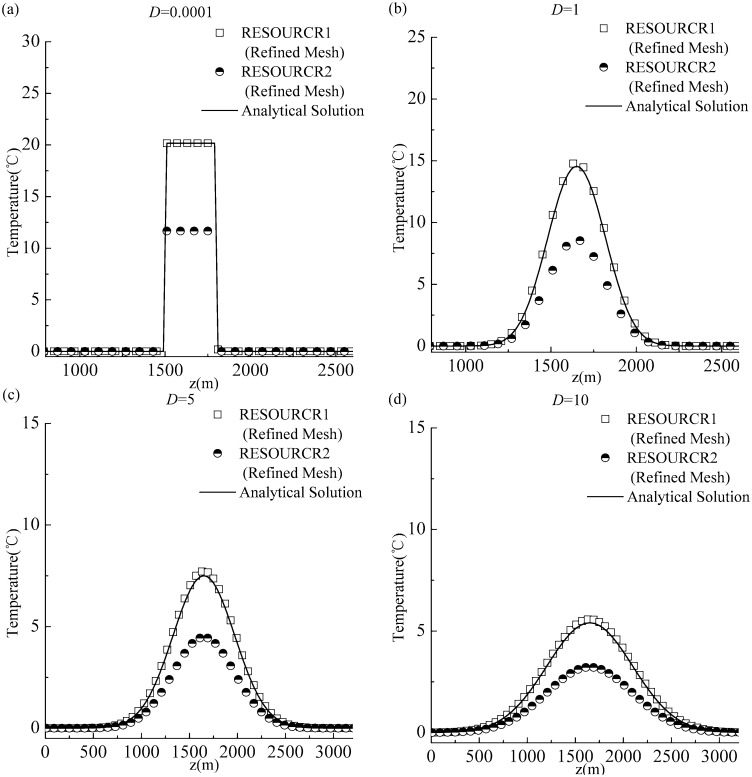
Comparison of simulation outcomes and analytical solutions for Experiment 2, using the refined mesh: (a) *D* = 0.0001; (b) *D* = 1; (c) *D* = 5; (d) *D* = 10.

The simulation results are shown in Fig 3 and the relative root mean square errors (RREs) are listed in Table 1. The RRE was generally smaller than 4% using the RESOURCE1 (the proposed high-order scheme for discretizing the source-sink term); the RRE was generally above 11% when using the RESOURCE2 (based on the pointwise method for discretizing the source-sink term). For all considered diffusion scenarios, ranging from the weak (D = 0.0001) to the strong (D = 10) diffusion, the results obtained using the proposed RESOURCE1 scheme [hollow squares in Fig 3(a)–3(d)] demonstrated better performance compared with the results obtained using the RESOURCE2. The simulated curve obtained using the RESOURCE2 distinctly deviated from the analytic curve, and the predicted results for the waveform and temperature peak values also deviated from their corresponding analytical results. These results suggest that, even when the second-order accurate Crank–Nicolson scheme is used for discretizing the diffusion part, a poor treatment of the source-sink term yields unsatisfactory results.

**Table 1:** RRE values for Experiment 2.

RRE	*D* = 0.0001	*D* = 1	*D* = 5	*D* = 10
RESOURCE1	3.5%	0.8%	1.2%	1.5%
RESOURCE2	11.4%	11.5%	15.2%	17.9%

The numerical deviations associated with the RESOURCE2 may originate from an inappropriate treatment of the diffusion term or of the source-sink term. The treatment of the diffusion term in Experiment 2 was identical to that in Experiment 1 (the Crank–Nicolson scheme was used for the diffusion term). However, comparing Figs 2 and 3 for the same space step (the refined mesh), the numerical solution calculated using the Crank–Nicolson scheme (CN) in Experiment 1 captured the diffusion process well (Fig 2); however, for Experiment 2, the numerical solution calculated using the RESOURCE2 (CN+ pointwise method), significantly differed from the numerical solution calculated using the RESOURCE1 (CN+ the proposed method) and the analytical solution (Fig 3). This observations support the assertion that the solution discrepancy reflects the improper treatment of the source-sink term, rather than the diffusion term. Therefore, we concluded that: even using a numerical scheme with higher-order accuracy and good stability to discretize the diffusion term, an inadequate discretization of the source-sink term will generate deviations between numerical and analytical solutions. This finding is consistent with the conclusions of Siviglia and Toro [13].

## Simulations of water temperature in the Guozheng Lake

The model and the proposed numerical method were used for performing simulations of water temperature in Guozheng Lake. Guozheng Lake is located in the city of Wuhan, province of Hubei (central China). Its geographical coordinates are 114° 21 ' E and 30° 33' N. The gross area of this lake is 11.3 km^2^, with the water level of 20 m. The average depth is 3.81 m and the maximal depth is 4.75 m. According to the available yearly observation data, the highest monthly temperature is 29.8°C (August) and the lowest monthly temperature is 4.8°C (January).

**Fig 4:**
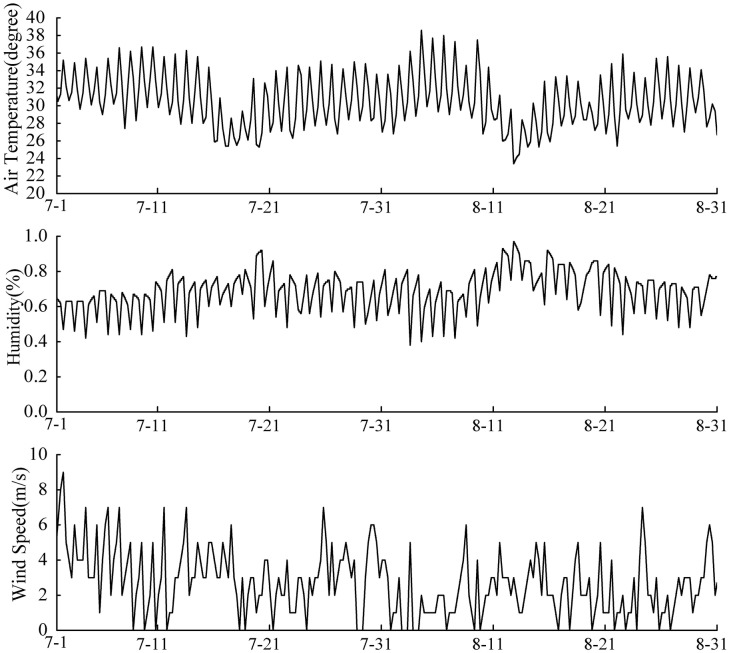
Meteorological data for the period from July 1 to August 31, 1978.

The recorded temperature data from July 1978 to August 1978 were used to validate the model and the numerical method. The year 1978 was the hottest among the 1976–2005 years. The observation period covers a significant range of lake thermal processes and yields large variations in the amplitude of water temperature, compared with other years. The meteorological data (including air temperature, relative humidity, and wind) were provided by the observation station of Lake Dong, located 3.5 km away from the lake. The original meteorological data used in this case are shown in Fig 4 and in S1 Table. Readers can access the website via “http://www.weather.com.cn” for more information on the meteorological data for Wuhan city. The observed data of water temperature were obtained from three observation ships and three fixed observation points. A 7151-2B conductor thermometer was used. The initial condition was the vertical distribution of water temperature on July 1, 1978. The sediment temperature during summer exhibited little change, so it was regarded as constant [31]. According to Cole [28], in this paper the typical value of the sediment temperature was defined as the average air temperature.

Here, we explain how to obtain α(t). A comparison was rendered between the average annual solar radiation and the average annual solar radiation from July to September 1978, which yielded a fit to α(*t*). The fitting function was α(*t*) = 0.03*(1+0.2sin0.0785*t*), with *t* = 0 corresponding to July 1, 1978. Thereafter, a comparison was performed between the observed solar radiation and the solar radiation calculated using the fit equation, for the period from July to September 1978. The calculated solar radiation curve was in an excellent agreement with the measured data. Thus, we believe that the functional form of α(*t*) can be used for estimating solar radiation. The detailed data on average annual solar radiation, the average annual solar radiation, and the observed solar radiation can be found in S2 Table. Although this has proven to be effective for simulating the summer period, the relatively simple functional form of α(*t*) cannot be guaranteed to be valid for all seasons. For example, we have tried to use this functional form to calculate the solar radiation during spring and the results were not satisfactory. Perhaps a more elaborate function or a piecewise mathematical function is required for describing other water bodies during other seasons.

In the case of Guozheng Lake, some consideration should be given to selecting the values of the spatial and temporal steps. If the selected vertical resolution is too high, the corresponding time step has to be very small to satisfy the CFL stability condition. This computation will require significant computing resources. Conversely, if the vertical resolution is too low, the model may not properly represent the actual vertical distribution of the water temperature. After several attempts, we found that when the lake was divided vertically into 10 cells (with Δz = 0.4 m), the vertical resolution converged to a value that correctly recognized the thermocline in most circumstances. In this case, the time step was Δt = 10 s.

### Simulation results and discussion

**Fig 5:**
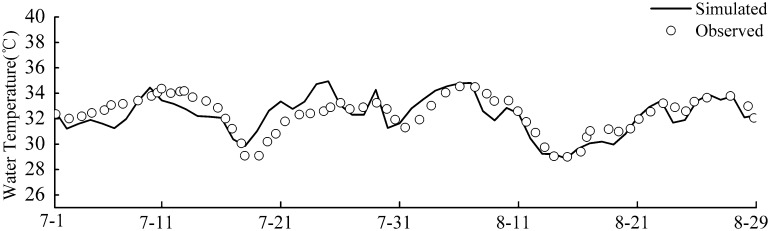
Simulated and observed daily average temperatures.

The simulated and the observed values of daily average temperature are shown in Fig 5. The simulation outcomes are in a good agreement with the observed data, accurately reflecting actual changes in the lake’s water temperature. The simulation results successfully capture the actual maximal temperature from August 5 to August 6. For the first few days, the simulation outcomes somewhat deviate from the actual data. This can be explained by noting that the model is in the “warm-up” period with respect to the numerical simulation; thus, some calculation errors are inevitable. After the “warm-up” period, the calculated values gradually approach the actual values.

**Fig 6:**
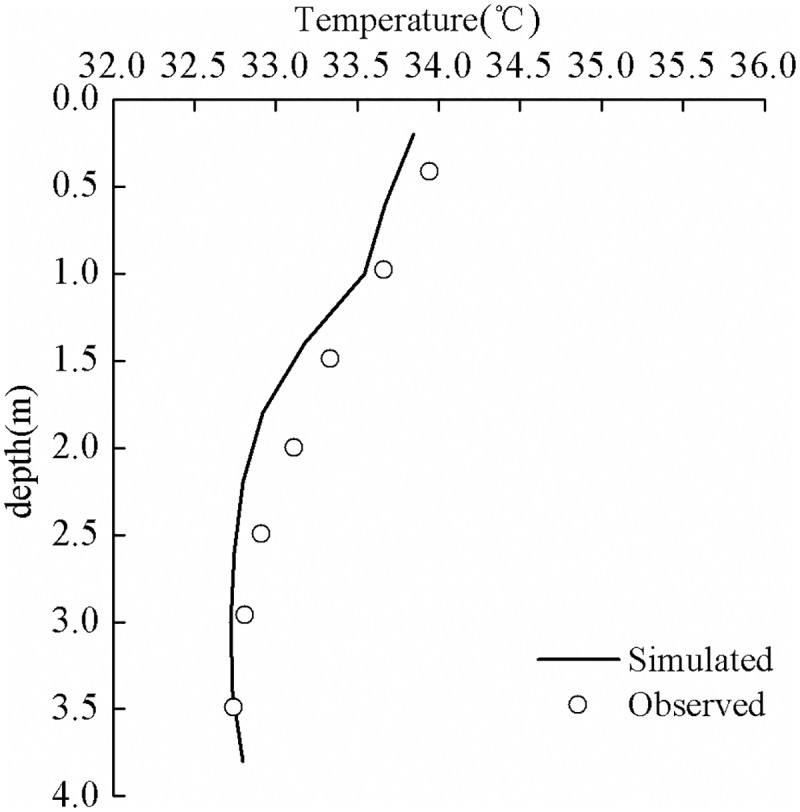
Simulated and observed water temperatures in the vertical direction, on July 13.

**Fig 7:**
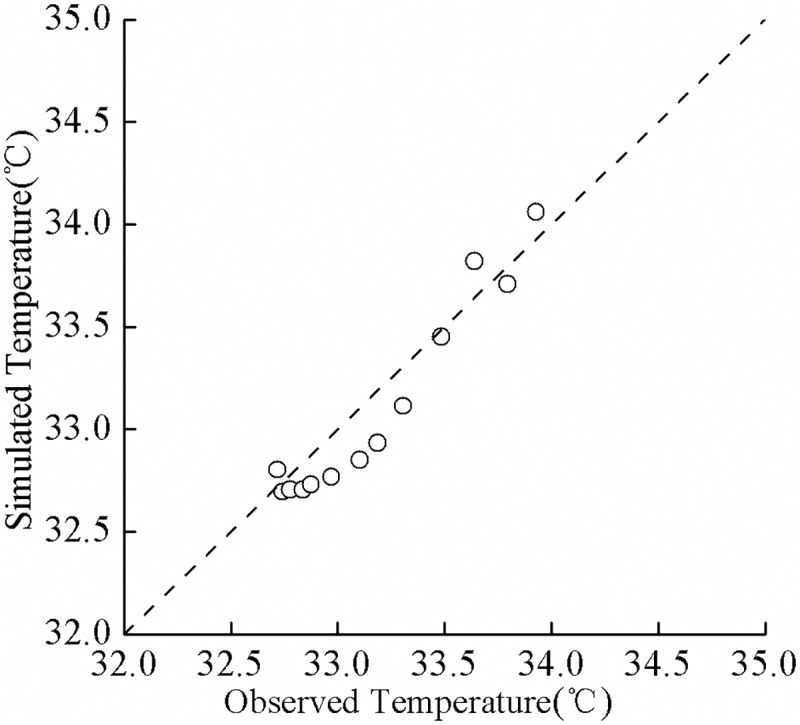
Correlation analysis of simulated and observed water temperatures.

July 13, 1978 was a fine day. The weather conditions were characterized by a typical high temperature and weak wind. According to the survey report of hydrologic characteristics pertaining to Guozheng Lake [32], the temperature difference between the surface and the bottom layers of water in the lake was 2°C (the measurement was performed near the center of the lake). Since this lake is a shallow urban lake and is usually well-mixed, the stratification phenomenon on July 13 was rare, which has great scientific research value. The simulated and the observed water temperatures in the vertical direction on July 13 are shown in Fig 6. According to the data in this figure, the simulation results were in a good agreement with real data. The simulation identified the distribution of the water temperature in the vertical direction and successfully predicted the major features of the positive temperature distribution. These features include the magnitudes of gradients in the vertical direction and the depth of the surface mixed layer, to name a few. For an overall assessment of the simulation outcomes in relation to actual data, Fig 7 shows the correlation between the simulated temperature and the observed temperature; the correlation coefficient R was bounded by 0.94. This further suggests that the simulation results are in a good agreement with actual data.

We also note that the simulated temperature in the vertical direction was somewhat smaller than the actual temperature (Fig 6, especially the thermocline temperature). This difference can be explained in terms of the wind patterns. On that day, wind directions were northeast, northwest, and southeast. However, there are trees in the northeast, northwest, and southeast bank corners of Guozheng Lake, and these trees provide sheltering, thereby decreasing the speed of wind at the lake. However, sheltering was not considered in the present model. Therefore, the input wind speed was higher than the actual speed, causing the calculated evaporation heat flux and the calculated convective heat transfer to be somewhat higher. These factors resulted in a lower simulated water temperature.

## Conclusion

In this paper, a novel numerical method was developed for solving a 1D vertical water temperature model. Using the operator-splitting method, the vertical 1D water temperature model was solved in two steps: (1) the source-sink term was discretized by a high order scheme, using an undetermined coefficient method; (2) the diffusion term was discretized by the Crank–Nicolson scheme. Two numerical tests were performed to validate the proposed method. Test results showed that the proposed numerical method is effective for solving equations with temperature-dependent source-sink terms. The results also demonstrated the importance of properly discretizing the source-sink term. Even when the diffusion term was solved using a high-accuracy and good-stability scheme, an inappropriate treatment of the source-sink term could yield numerically inaccurate solutions. Finally, the model and the proposed numerical method were used for simulating the temperature distribution in Guozheng Lake, and the simulation results were in a good agreement with real data.

## Supporting information

S1 Table Original data of meteorological input. (XLSX)

S2 Table Original data of average annual solar radiation and average annual water temperature from July to September as well as the observed solar radiation from July to September in 1978. (XLSX)
